# Supplementary material for: A Simple Differentiation Protocol for Generation of Induced Pluripotent Stem Cell-Derived Basal Forebrain-Like Cholinergic Neurons for Alzheimer’s Disease and Frontotemporal Dementia Disease Modeling
Source: Cells. 2020 Sep 2;9(9):2018. doi: 10.3390/cells9092018 (PMC7564334; doi:10.3390/cells9092018)
Supplement: Supplementary file 1 [file cells-09-02018-s001.pdf]

Article, *Special Issue "Induced Pluripotent Stem Cells in Neurodegenerative Diseases: Application for Therapy and Disease Modeling"*

A simple differentiation protocol for generation of induced pluripotent stem cell-derived basal forebrain cholinergic neurons for Alzheimer's disease and frontotemporal dementia disease modeling

Supplemental information

Method 1. Reprogramming and characterisation of MBE2960 healthy control iPSC line

The iPSCs were generated using skin fibroblasts obtained from subjects over the age of 18 years by episomal method as described [40]. Briefly, reprogramming was performed on passage 8-10 fibroblasts by nucleofection (Lonza Amaxa Nucleofector) with episomal vectors expressing *OCT4*, *SOX2*, *KLF4*, *L-MYC*, *LIN28* and shRNA against *p53* [41] in feeder- and serum- free conditions using TeSR-E7 medium (Stemcell Technologies). Subsequently, reprogrammed colonies were manually dissected to establish clonal cell lines [42]. Three clones were assessed for pluripotency markers via immunocytochemistry (Figure S1A). The iPSC line was expanded and characterised. Embryoid bodies were obtained as described [43] and using tri-lineage differentiation kit (Stemcell Technologies). Germ layer differentiation was assessed by immunochemistry (Figure S1B). Copy number variation (CNV) analysis of original fibroblasts and iPSCs from MBE2960 (Figure S1C) was performed using Illumina HumanCore Beadchip arrays as we described [40]. CNV analyses were performed using PennCNV and QuantiSNP with default parameter settings [44,45]. Chromosomal aberrations were deemed to involve at least 10 contiguous single nucleotide polymorphisms (SNPs) or a genomic region spanning at least 1MB [44,45]. The B allele frequency (BAF) and the log R ratio (LRR) were extracted from GenomeStudio (Illumina) for representation (Figure S1D).

Method 2. *Reprogramming and characterization of a late-onset (sporadic) Alzheimer's disease iPSC line UOWi006-A and a frontotemporal dementia / amyotrophic lateral sclerosis (FTD/ALS C9orf72 expansion) UOWi008-A iPSC line*

A skin biopsy was obtained from an 83-year-old female with sporadic Alzheimer's disease (*APOE* ε4/4 genotype) (RB7-11 clone) and a 66-year-old female patient with diagnosed with frontotemporal dementia / amyotrophic lateral sclerosis (FTD/ALS, caused by a *C9orf72* expansion) (C-10 clone), following informed consent from the donor. The study was approved by the University of Wollongong Human Ethics Committee (HE13/299). Dermal fibroblasts were cultured at 37°C and 5% CO_2 in Dulbecco's Modified Eagle Medium F12 (DMEM/F12) supplemented with 1x Non-Essential Amino Acids (Thermo Fisher Scientific) and 10% foetal bovine serum (Interpath). Fibroblasts were reprogrammed using Stemgent microRNA-Enhanced mRNA Reprogramming kit (Stemgent) with pluripotency transcription factors Oct4, Klf4, Sox2, c-Myc, Lin28 and Nanog, following the manufacture's protocol. Prior to reprogramming, Pluriton reprogramming medium was conditioned using new-born human foreskin fibroblasts (Global Stem), as per the reprogramming protocol. Spontaneous iPSC colonies were isolated on day 14 for expansion into individual iPSC lines (clones). Established iPSC clones were maintained at 37°C and 5% CO_2 on Matrigel-coated plates in TeSR-E8 and were split 1:5 using dispase on reaching 70% confluence.

The first iPSC colonies appeared on day 10 and were isolated on day 14 for expansion and characterisation, with clone 11 selected following confirmation of pluripotency. The iPSC colonies had normal morphology (Figure S2A) and karyotype (Figure S2B), with no abnormalities detected in 15 cells at 400 bands per haploid set. The identity of iPSCs was authenticated against its parental fibroblast line via short tandem repeat (STR) profiling. Transcription of endogenous pluripotency genes *NANOG* and *POU5F1* increased by 210 and 1,300-fold, respectively, in comparison to parental fibroblasts (Figure S2C) and immunocytochemical analysis demonstrated expression of pluripotency markers Oct4, SSEA-4 and TRA-1-60 (Figure S2D). Differentiation potential into the three-germ layers was confirmed using the hPSC Scorecard assay (Figure S2E).

Characterisation of iPSC colonies via karyotyping, STR analysis, qPCR, immunofluorescence and hPSC Scorecard analysis was performed as previously described [15].

List of genes

Table S1. List of genes analysed in Nanostring for iPSC and BFCN cultures.

Gene name	Approved name	Accession	Position	Category
AARS	Alanyl-tRNA synthetase	NM_001605.2	836-935	Housekeeper
ACHE	Acetylcholinesterase	NM_000665.3	1058-1157	BFCN
ALDOC	Aldolase, fructose-bisphosphate C	NM_005165.2	261-360	Astrocytes
ASB7	Ankyrin repeat and SOCS box containing 7	NM_024708.3	1281-1380	Housekeeper
ASCL1	Achaete-scute homolog 1	NM_004316.3	1651-1750	Neuronal progenitor
CCDC127	Coiled-coil domain-containing 127	NM_145265.2	295-394	Housekeeper
CHAT	Choline O-acetyltransferase	NM_020549.4	1106-1205	BFCN
SLC5A7 (CHT1)	Solute carrier family 5 member 7 (choline transporter 1)	NM_021815.2	956-1055	BFCN

CNOT10	CCR4-NOT transcription complex subunit 10	NM_001256741.1	1963-2062	Housekeeper
SLC6A3 (DAT1)	Solute carrier family 6 member 3 (dopamine transporter 1)	NM_001044.3	1549-1648	Neuronal
DLX1	Distal-less homeobox 1	NM_001038493.1	1336-1435	Neuronal progenitor
DLX2	Distal-less homeobox 2	NM_004405.3	591-690	Neuronal progenitor
SLC1A3 (EAAT1)	Solute carrier family 1 member 3 (glial high affinity glutamate transporter 1)	NM_004172.4	559-658	Astrocytes
EID2	EP3000-interacting inhibitor of differentiation 2	NM_153232.3	566-665	Housekeeper
EMX1	Empty-spiracles homeobox 1	NM_004097.2	1747-1846	Neuronal progenitor
FOXG1	Forkhead box protein G1	NM_005249.3	1401-1500	BFCN progenitor
GAD2	Glutamate decarboxylase 2	NM_000818.2	1246-1345	Neuronal
GRIA1	Glutamate ionotropic receptor AMPA type subunit 1	NM_000827.3	2841-2940	Neuronal
GRIA2	Glutamate ionotropic receptor AMPA type subunit 2	NM_001083620.1	866-965	Neuronal
GRIN1	Glutamate ionotropic receptor NMDA type subunit 1	NM_000832.5	1291-1390	Neuronal

ISL1	Insulin Enhancer protein (ISL) LIM homeobox 1	NM_002202.2	1376-1475	BFCN progenitor
LHX8	LIM homeobox 8	NM_001001933.1	1301-1400	BFCN progenitor
MAP2	Microtubule associated protein 2	NM_031845.2	5171-5270	Neuronal
MTO1	Mitochondrial tRNA translation optimization 1	NM_133645.2	1466-1565	Housekeeper
NANOG	Nanog homeobox	NM_024865.2	1101-1200	Pluripotency
NFIA	Nuclear factor I A	NM_001134673.2	1086-1185	Astrocytes
NGFR	Nerve growth factor receptor	NM_002507.3	2731-2830	BFCN
NKX2-1	NK2 homeobox 1	NM_003317.3	2012-2111	BFCN progenitor
NTRK1 (TRKA)	Neurotrophic receptor tyrosine kinase 1	NM_001012331.1	1366-1465	BFCN
PAX6	Paired box 6	NM_000280.3	1174-1273	Neuronal progenitor
POUF51	POU class 5 homeobox 1	NM_002701.4	1226-1325	Pluripotency
DLG4 (PSD95)	Disc large MAGUK scaffold protein 4 (post-synaptic density protein 95)	NM_001365.3	2461-2560	Neuronal
RABEP2	Rabaptin, RAB GTPase-binding effector protein 2	NM_024816.2	1783-1882	Housekeeper

S100B	S100 calcium binding protein B	NM_006272.2	305-404	Astrocytes
SOX1	SRY-box 1	NM_005986.2	1496-1595	Neuronal progenitor
SUPT7L	SPT7 like, STAGA complex gamma subunit	NM_014860.2	1171-1270	Housekeeper
SYN1	Synapsin I	NM_006950.3	566-665	Neuronal
TADA2B	Transcriptional adaptor 2B	NM_152293.2	1589-1688	Housekeeper
TH	Tyrosine hydroxylase	NM_000360.3	1307-1406	Neuronal
TUBB3	Tubulin beta 3 class III	NM_006086.2	1538-1637	Neuronal
SL18A3 (VACHT)	Solute carrier family 18 member A3 (vesicular acetylcholine transporter)	NM_003055.2	1651-1750	BFCN
ZNF324B	Zinc finger protein 324B	NM_207395.2	2821-2920	Housekeeper

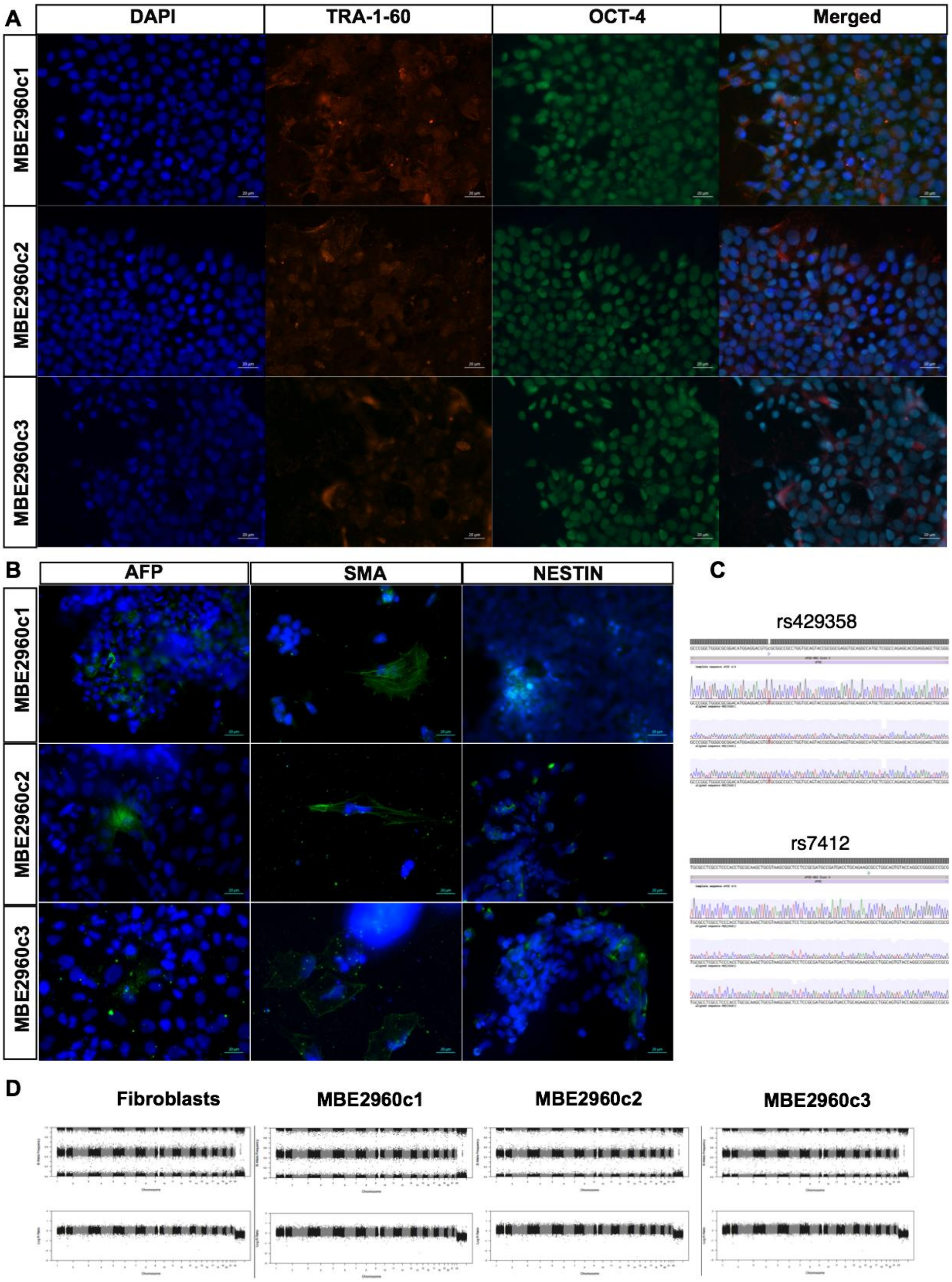

Figure S1. Confirmation of pluripotency of iPSCs MBE2960 healthy control. A. iPSC colonies were positive for the immunocytochemical staining for pluripotency markers TRA-1-60 (Red) and Oct-4 (Green) with DAPI (Blue). B. iPSCs were differentiated into the three germ layers and confirmed via immunocytochemical staining for AFP (Endoderm), SMA (Mesoderm) and Nestin (Ectoderm) with DAPI. C. Copy number variation (CNV) analysis of original fibroblasts and iPSCs from MBE2960 D. Representation of the B allele frequency (BAF) and the log R ratio (LRR) of the fibroblasts and the iPSC clones. Scale bars = 20 μm.

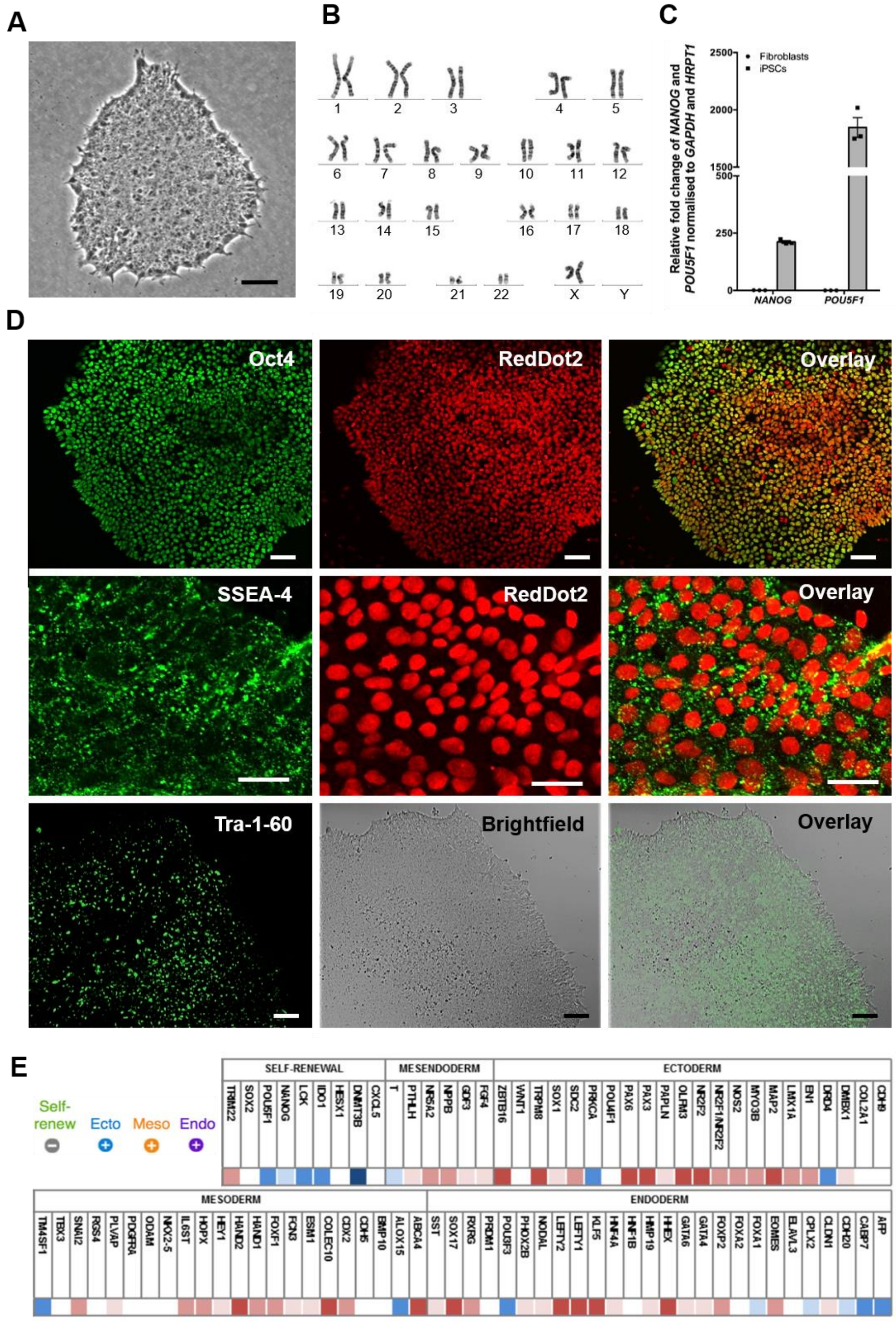

Figure S2. Confirmation of pluripotency and three germ layer differentiation of iPSCs RB7-11 late-onset Alzheimer's disease (UOWi006-A). iPSC colonies showed normal A. morphology and B. karyotype. C. RT-qPCR analysis demonstrated fold change in expression of pluripotency transcription factors *NANOG* and *POU5F1* to parental fibroblasts. D. Immunocytochemical staining for pluripotency markers Oct4, SSEA-4 and TRA-1-60 (green) with RedDot2 nuclear marker (red) or brightfield. E. Three germ layer differentiation showed fold change (fc) in expression for specific sets of genes for self-renewal and ectoderm, mesoderm and endoderm germ layers based on TaqMan hPSC Scorecard. Data points on graph represent technical replicates on RT-qPCR. Scale bars = 50 μm.

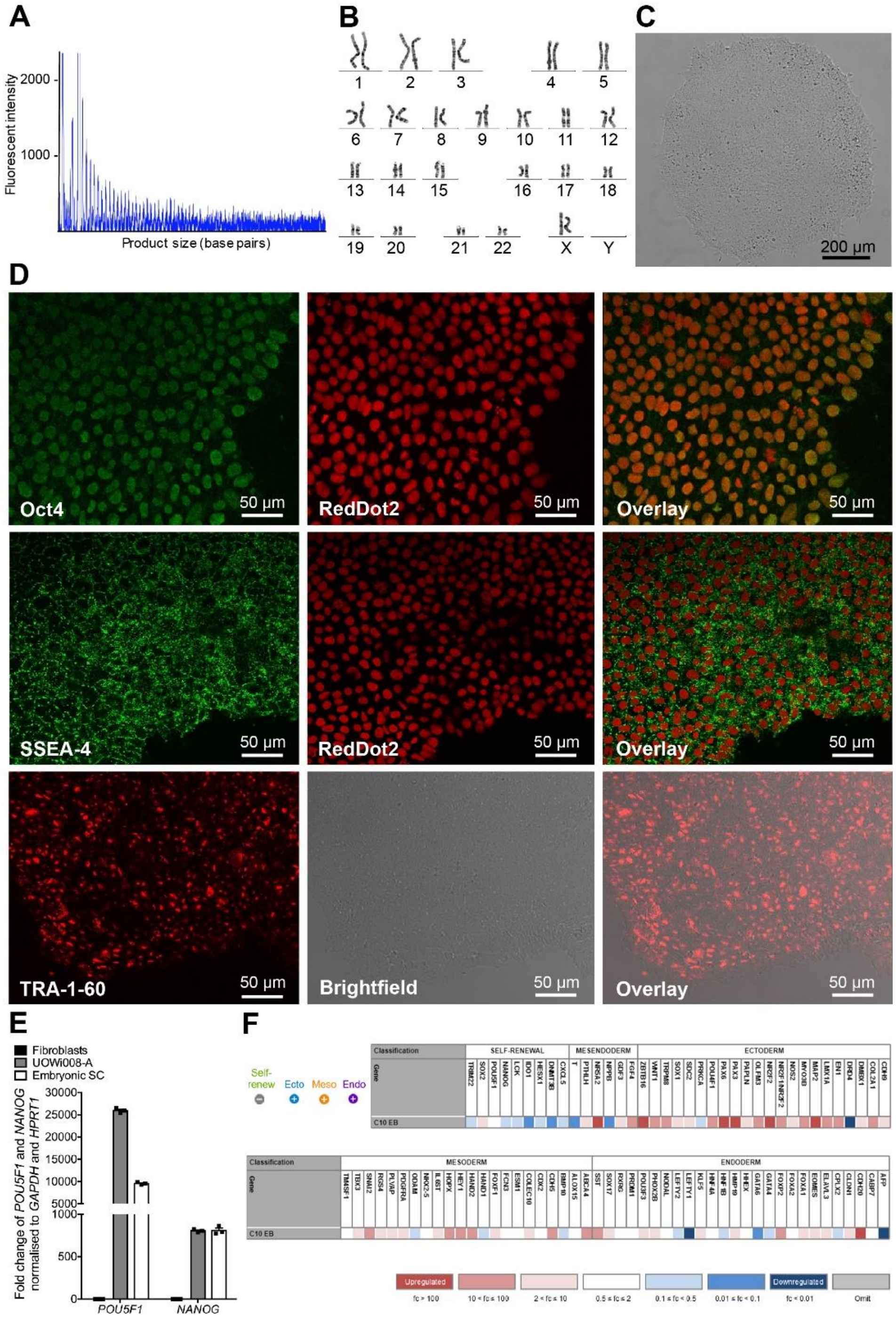

Figure S3. Confirmation of pluripotency and three germ layer differentiation of iPSCs C-10 FTD/ALS (UOWi008-A). The hexanucleotide repeat in C9orf72 was genotyped using the repeat primed polymerase chain reaction (PCR) method described by Renton et al. (2011), with a pathogenic expansion defined as more than 30 repeat units. Fragment length analysis was performed by Macrogen Inc (South Korea) using the ABI 3730XL DNA analyser (Applied Biosystems, CA, USA) and data were analysed using Peak Scanner 2 (Life Technologies) A. Cells showed normal karyotype B. and morphology C. Immunocytochemical staining for pluripotency markers Oct4, SSEA-4 and TRA-1-60 (green) with RedDot2 nuclear marker (red) or brightfield D.. RT-qPCR analysis demonstrated fold change in expression of pluripotency transcription factors *NANOG* and *POU5F1* to parental fibroblasts. E. Three germ layer differentiation showed fold change (fc) in expression for specific sets of genes for self-renewal and ectoderm, mesoderm and endoderm germ layers based on TaqMan hPSC Scorecard F. Data points on graph represent technical replicates on RT-qPCR. Scale bars = 50 µm.

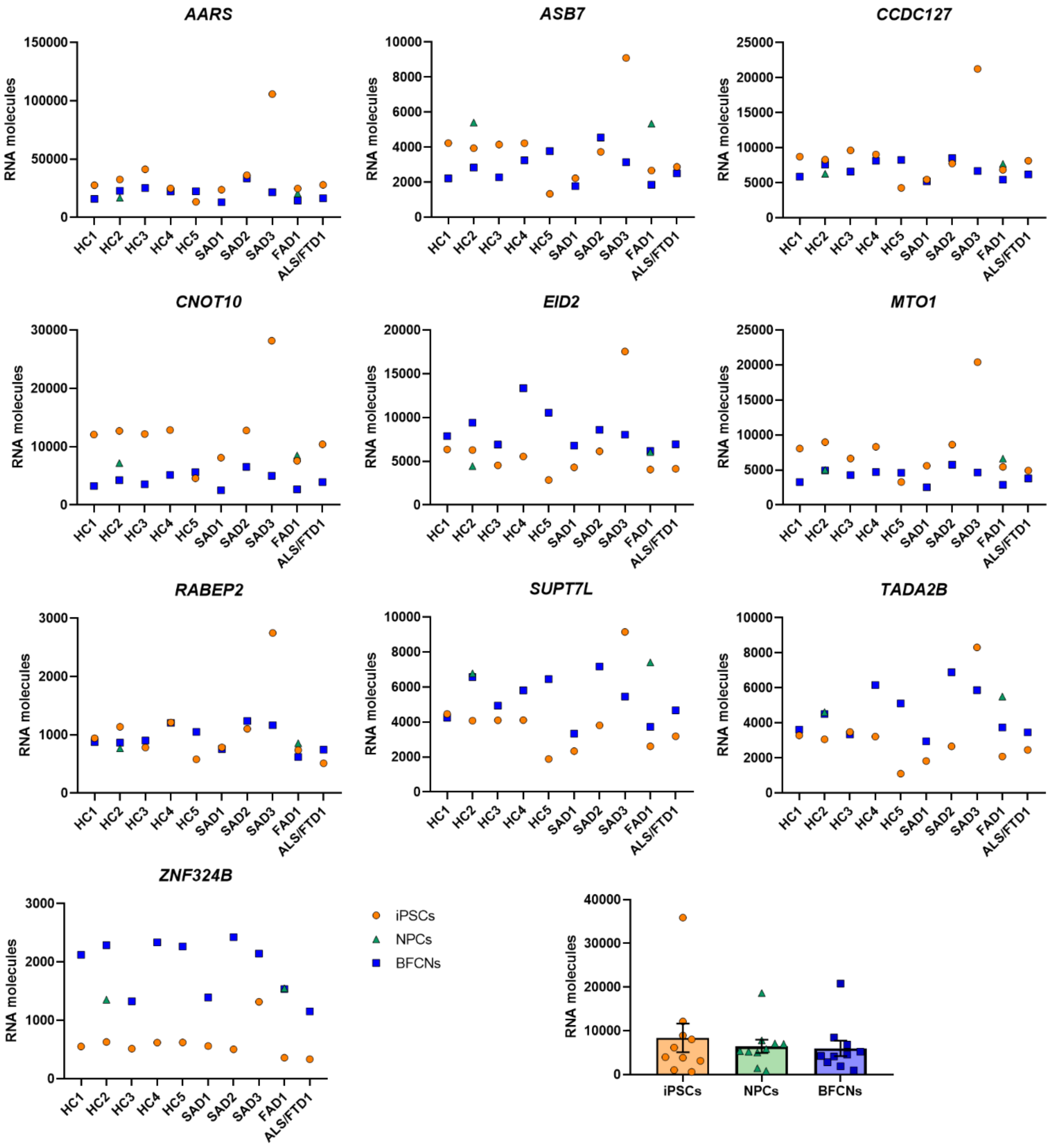

Figure S4. RNA molecule count of housekeeper genes. iPSCs, NPCs and BFCNs samples were analysed by nCounter (Nanostring) and results are shown as RNA molecule count after internal quality control and normalisation to a reference sample used on the PlexSet. The ten housekeeper genes *AARS*, *ASB7*, *CCDC127*, *CNOT10*, *EID2*, *MTO1*, *RABEP2*, *SUPT7L*, *TADA2B* and *ZNF324B* were analysed. The overall housekeeper expression between iPSCs, NPCs and BFCNs shows no significant difference.

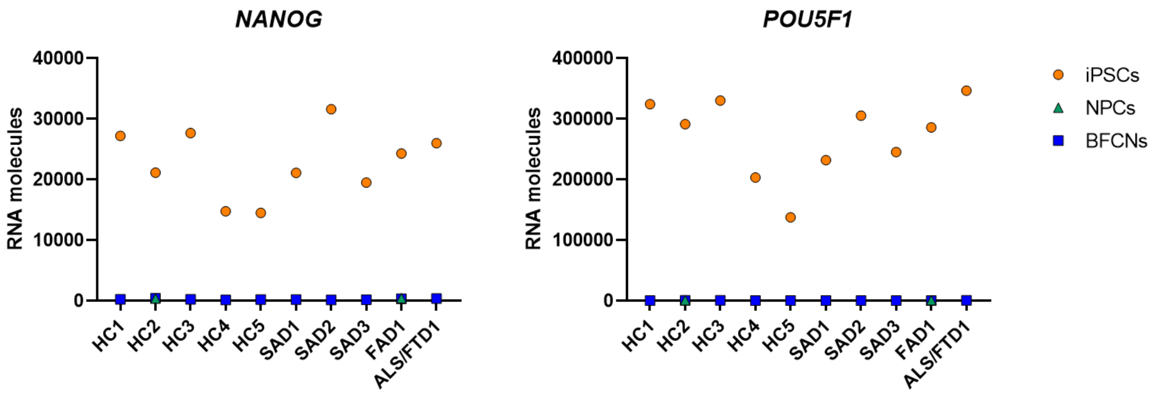

Figure S5. RNA molecule count of pluripotency markers. iPSCs, NPCs and BFCNs samples were analysed by nCounter (Nanostring) and results are shown as RNA molecule count after normalisation of total amount of RNA molecules to the housekeeper genes. The pluripotency markers *NANOG* and *POU5F1* were analysed.

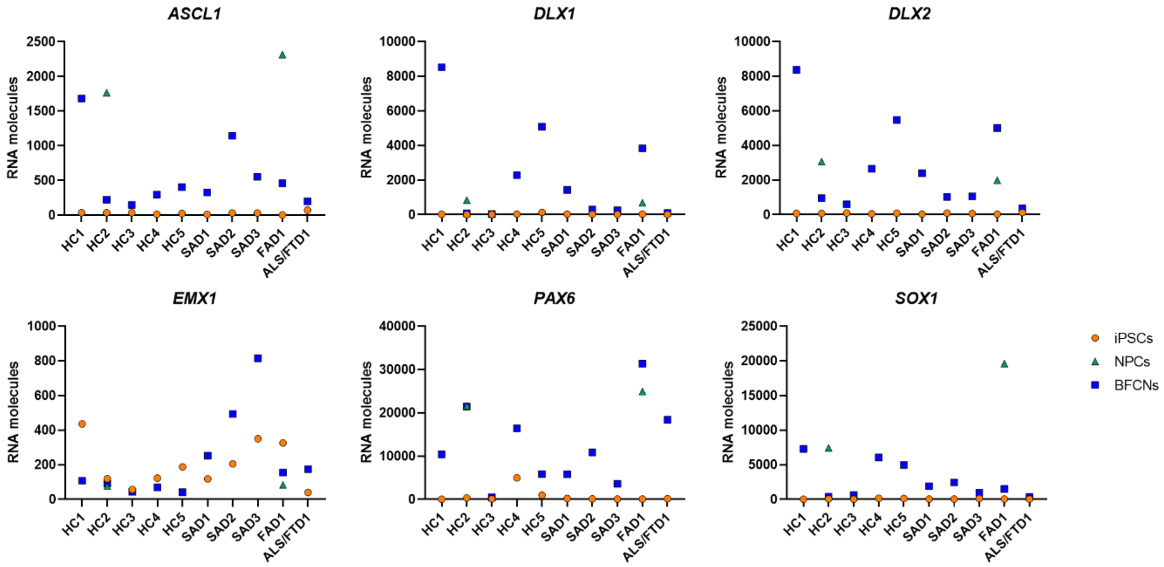

Figure S6. RNA molecule count of developmental markers. iPSCs, NPCs and BFCNs samples were analysed by nCounter (Nanostring) and results are shown as RNA molecule count after normalisation of total amount of RNA molecules to the housekeeper genes. The developmental markers *ASLC1*, *DLX1*, *DLX2*, *EMX1*, *PAX6* an *SOX1* were analysed.

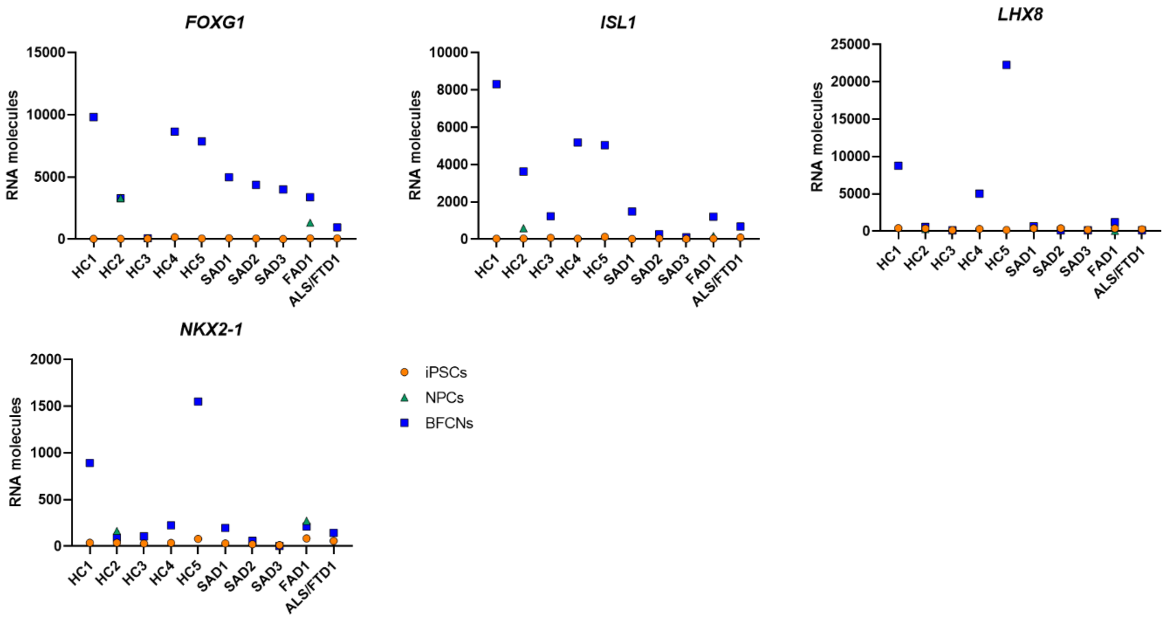

Figure S7. RNA molecule count of cholinergic developmental markers. iPSCs, NPCs and BFCNs samples were analysed by nCounter (Nanostring) and results are shown as RNA molecule count after normalisation of total amount of RNA molecules to the housekeeper genes. The cholinergic developmental markers *FOXG1*, *ISL1*, *LHX8* and *NKX2-1* were analysed.

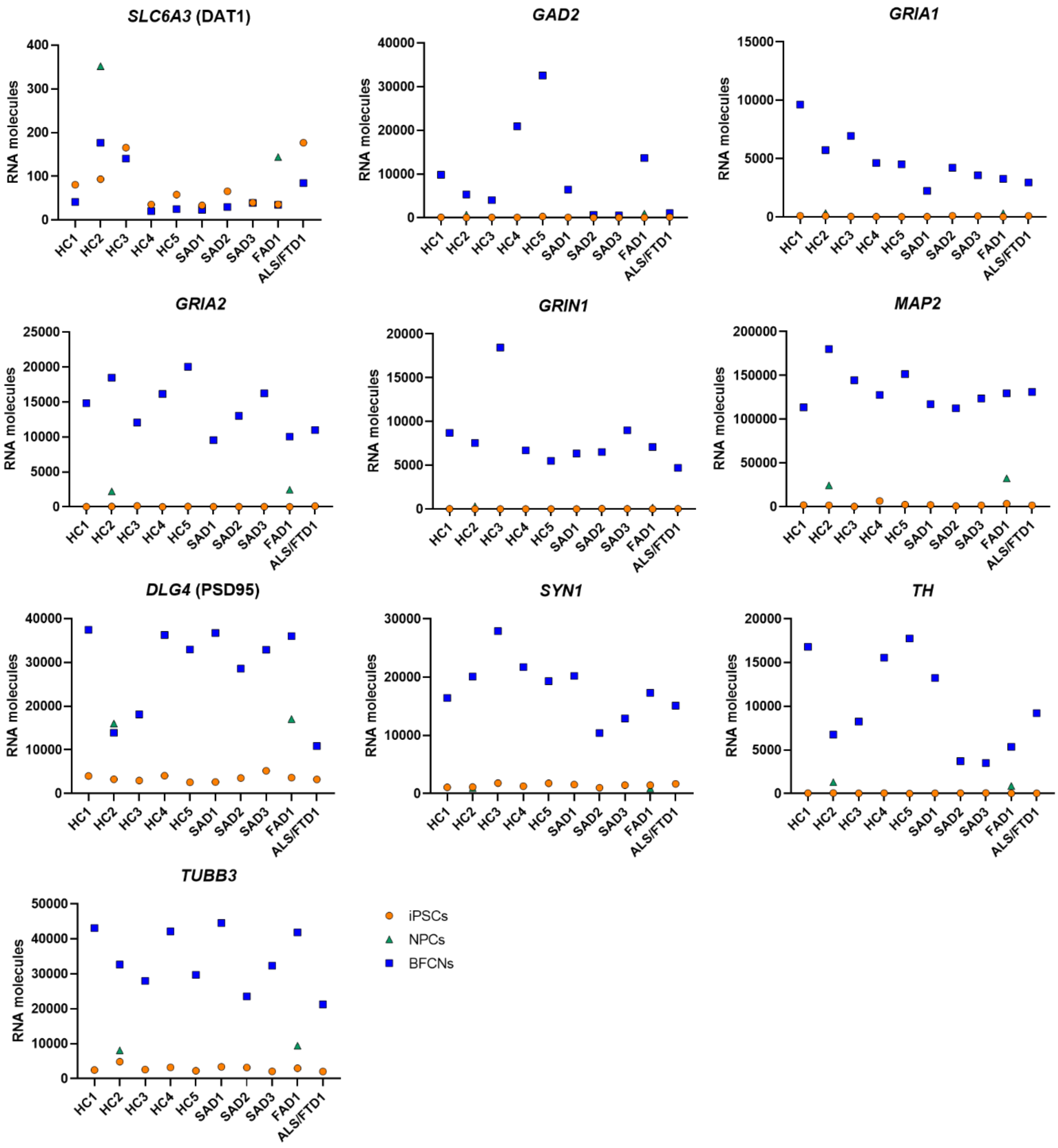

Figure S8. RNA molecule count of neuronal markers. iPSCs, NPCs and BFCNs samples were analysed by nCounter (Nanostring) and results are shown as RNA molecule count after normalisation of total amount of RNA molecules to the housekeeper genes. The neuronal markers *SLC6A3* (DAT1), *GAD2*, *GRIA1*, *GRIA2*, *GRIN1*, *MAP2*, *DLG4* (PSD95), *SYN1*, *TH* and *TUBB3* were analysed.

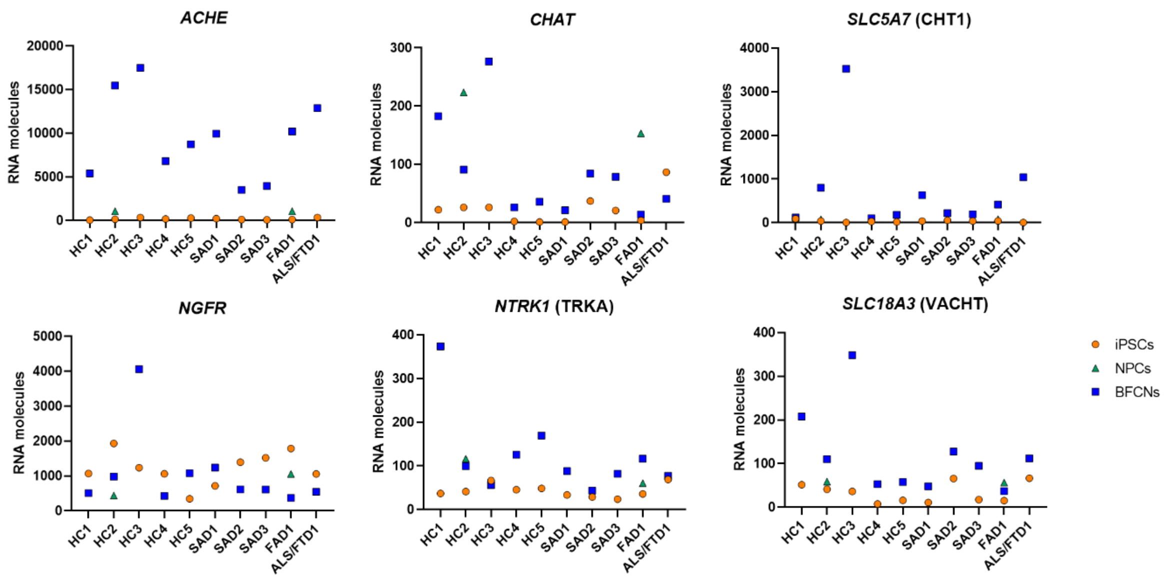

Figure S9. RNA molecule count of cholinergic neuron markers. iPSCs, NPCs and BFCNs samples were analysed by nCounter (Nanostring) and results are shown as RNA molecule count after normalisation of total amount of RNA molecules to the housekeeper genes. The cholinergic neuronal markers *ACHE*, *CHAT*, *SLC5A7* (CHT1), *NGFR*, *NTRK1* (TRKA) and *SLC18A3* (VACHT) were analysed.

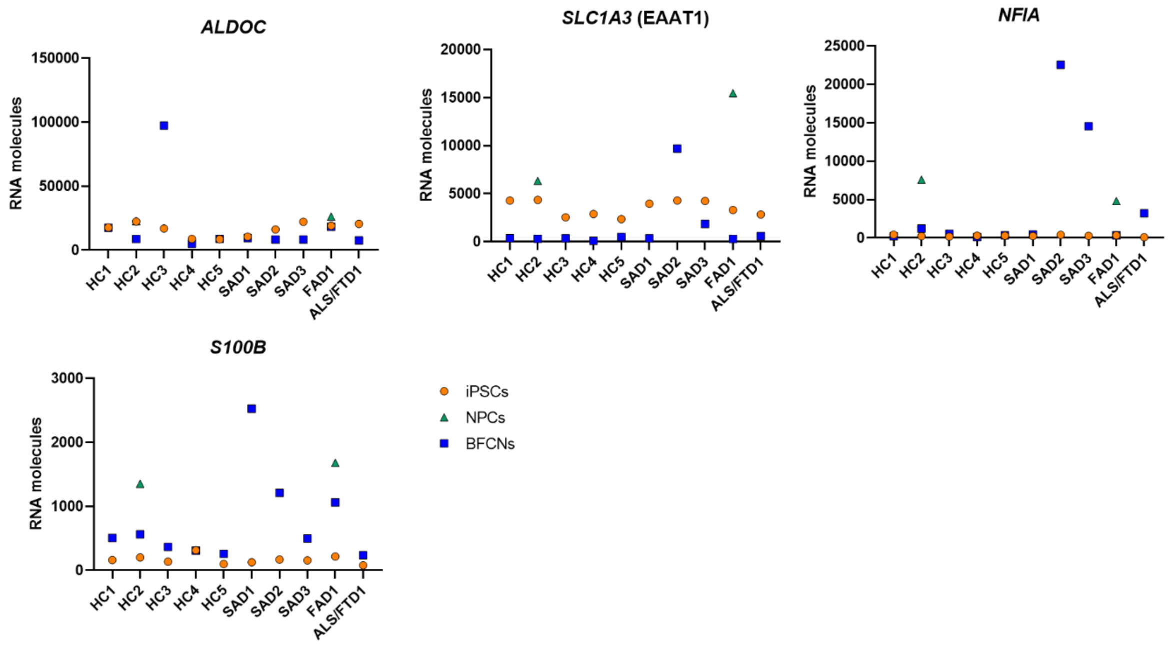

Figure S10. RNA molecule count of astrocytic markers. iPSCs, NPCs and BFCNs samples were analysed by nCounter (Nanostring) and results are shown as RNA molecule count after normalisation of total amount of RNA molecules to the housekeeper genes. The astrocytic markers *ALDOC*, *SLC1A3* (EAAT1), *NFIA* and *S100B* were analysed.

© 2020 by the authors. Submitted for possible open access publication under the terms and conditions of the Creative Commons Attribution (CC BY) license (http://creativecommons.org/licenses/by/4.0/).
